# Vascular injuries of the extremities are a major challenge in a third world country

**DOI:** 10.1186/s13032-015-0027-0

**Published:** 2015-07-30

**Authors:** Fahad H. Khan, Kamal M. Yousuf, Anel R. Bagwani

**Affiliations:** General Surgery, Civil Hospital, Baba-e-Urdu Road, Karachi, 75200 Pakistan; Vascular Surgery, Liaquat National Hospital, Stadium Road, Karachi, 74800 Pakistan; General Surgery, Liaquat National Hospital, Stadium Road, Karachi, 74800 Pakistan

**Keywords:** Trauma, Vascular injuries, Extremities, Developing countries

## Abstract

**Background:**

Traumatic vascular injuries of the extremities are a major challenge especially in the third world countries. These injuries are mostly due to poor traffic laws, street crimes, firearms and blast associated injuries. We therefore would like to share our 10 years of experience in dealing with vascular injuries in Pakistan.

**Methods:**

This was a retrospective observational study conducted in the department of vascular surgery of Liaquat National Hospital, Karachi, Pakistan. Patients’ records were retrieved from the department and were reviewed. Cases with vascular injuries of upper and lower limb that presented with signs of salvageable limb and presented within 12 hours of injury were included in the study. Patients with more than 12 hours of presentation and in whom primary amputation was done, were excluded from the study.

**Results:**

There were 328 patients who presented with vascular injuries of the extremities that fell in the inclusion criteria. Limb salvage rate was 41 %, whereas 30-days perioperative mortality was 5.48 %. The major cause of limb loss was delay in presentation of more than 8 h of injury. Major vessels involved were popliteal artery (41.76 %), followed by femoral artery (27.43 %).

**Conclusion:**

Vascular injuries are becoming a major contributor of limb loss in third world countries due to violence, terrorism and unavailability of vascular facilities. This morbidity can be reduced by improving law and order situation, evolving an effective emergency ambulatory system and with better training and provision of vascular services in remote areas so that the delay factor can be reduced.

## Background

Vascular trauma of the extremities are very common in third world countries. Vascular injuries secondary to penetrating trauma remains a significant cause of morbidity and mortality in both civilian and military population. These injuries constitute about 3 % of civilian injuries and around 7 % of combat associated trauma [1]. In developing countries it is mostly due to motor vehicle accidents, street crimes, improvised explosive devices and industrial accidents. Liaquat National Hospital is one of the largest private sector tertiary care centre in Karachi, Pakistan and is one with busy trauma units with vascular surgery expertise in Karachi. Many victims of vascular trauma present to this centre not only from Karachi but from other areas of Pakistan and neighbouring countries like Iran, Iraq and Afghanistan. Being a developing country, our country lacks effective emergency ambulatory facilities, especially for trauma patients which is a major contributing factor for delayed presentation of these patients to tertiary care centers. Early recognition of vascular injuries is essential for prompt management. Delay may cause irreversible ischemic injuries which may result in impaired limb function or limb loss [2]. Through this study we would like to share our 10 years of experience of dealing vascular injuries of the extremities in a tertiary care centre of a third world country like Pakistan, where lack of basic facilities and unavailability of expertise services remain a common problem.

## Methods

This was a retrospective study of 10 years between January, 2002 and January, 2012 conducted in department of Vascular Surgery, Liaquat National Hospital, Karachi, Pakistan. Case records of all the patients presenting with traumatic injuries were reviewed for demographic profile, including age and gender, mechanism and location of injury, repair techniques, limb salvage rate and complication rates. The data was entered and analysed on Statistical Package for Social Sciences (SPSS) version 16 for frequencies and co-relations with significant *p*-value of <0.05. This study has been approved by the institute’s ethical review committee.

Salvageable limb was defined as the limb with signs of viability (warm, positive distal pulses, >90 % oxygen saturation and intact neurological signs), with repairable soft tissues and skeletal injuries. The inclusion and exclusion criteria were set as:

### Inclusion criteria

- All the patients who presented with vascular injuries of extremities with or without associated orthopaedics and soft tissue injuries.
- Presentation within 12 h with signs of viabilities.
- Patients who presented with salvageable limbs with duration of >12 h from the injury.

### Exclusion criteria

- Associated head injuries or major injuries to abdomen necessitating urgent attention first.
- Non-salvageable limb (with major tissue loss/mangled limbs).
- Delayed presentation i.e. after 12 h of injury with no signs of viability.

## Results

**Fig. 1:**
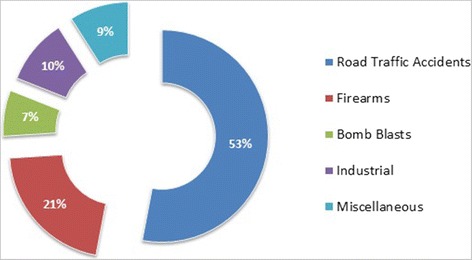
Etiologies of vascular injuries (n = 328)

We studied 412 patients who presented with vascular injuries of extremities during the past 10 years, but 328 cases met our inclusion criteria. There were 90.2 % (296) males and 9.8 % (32) females with mean age of 43 ± 7 years. Presentation was early, within 8 h, in 79.2 % (260) patients. The mechanism of trauma was road traffic accidents (RTA) (53 %), gunshots/firearms (21 %) and bomb blasts (7 %) (Fig. 1). Pre-operative angiography was performed in cases with absent hard signs of vascular injuries (absent pulses, bruit or palpable thrill, active haemorrhage, expanding hematoma or distal ischemia) in 9.4 % (31) cases, among which 58 % (18) had computed topographic angiography (CTA) and 42 % (13) had conventional angiography.

**Fig. 2:**
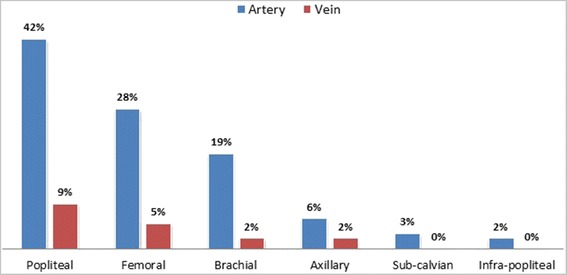
Pattern of vascular involvement of the extremities (n = 328)

The limb salvage rate was 41 %; however in 59 % (194) cases, where the limb could not be saved, 56.4 % (109) (*p*-value: 0.03) cases had a delay in presentation of more than 8 hours duration from the time of injury while others developed local complications even after vascular repair (Table 1). Sixty two percent (119) (*p*-value: 0.06) had limb loss from blunt injuries and 38 % (75) (*p*-value: 0.18) had penetrating injuries. The vasculature in lower extremity was involved more commonly as compared to upper extremity, as popliteal artery in 41.7 % and femoral artery in 27.4 % (Fig. 2) were top on the list. Associated bone injuries were found in 36.5 % (116) patients, 25.5 % (81) had muscle/tendon injuries and 21.1 % (67) had nerve injuries.

**Table 1:** Post-operative complications in traumatic patients after vascular repair (n = 139)

	% (N)
LOCAL	
Wound Infection	13.1 (43)
Graft Thrombosis	6.4 (21)
Graft Infection	3.9 (13)
Haemorrhage	2.4 (8)
SYSTEMIC	
Renal Failure	9.7 (32)
Respiratory failure	3.6 (12)
Sepsis	2.4 (8)
Cerebrovascular accident	0.6 (2)

The surgical repair of the arteries was with end to end primary anastomosis in 9.7 % (32) cases. Autologous saphenous vein grafts from opposite limb were used in 53.1 % (169) and polytetraflouroethylene (PTFE) grafts in 39.9 % (127) cases. In 8.5 % (28) patients venous repair was carried out which included femoral, popliteal and sub-clavian veins, while rests of the venous injuries were ligated. No shunts were placed between the two ends of vessels during the surgical repairs. Initial soft tissue cover for the repaired vessels was done in 18.9 % (62) cases.

Out of 67 patients who had associated nerve injuries, primary nerve repair was done in 59.7 % (40) cases, while in 40.2 % (27) cases the nerve was tagged with non-absorbable suture for secondary repair. Bony injuries, in 116 cases, were managed by orthopedic surgeons using bone fixation in 74.1 % (86) and splints in 25.8 % (30) cases. Fasciotomies were done in 34.5 % (110) cases to prevent and relief compartment syndrome, which were subsequently closed with primary closure in 32.7 % (36) and was covered with skin grafts in 67.2 % (74) patients. The main vascular complication was wound infection in 13.1 % (43) of the cases and the main non-vascular complication was acute renal failure in 9.7 % (32) (Table 1).

### Outcomes

Post-operative vascular analysis was done by physical examination of distal pulses, oxygen saturation at toe level in all cases. Ankle Brachial Pressure Index (ABPI) was measured where it was possible. Post-operatively all patients were prescribed low molecular weight heparin for deep venous thrombosis prophylaxis, till their mobilization out of bed. Hospital stay varied from 5–35 days depending upon the severity of injury inclusive of bone and soft tissue injuries. Follow-up was done according to the specialties involved. In particular for vascular injuries follow up was done on 1^st^, 2^nd^ and 4^th^ week. Long term follow-up was lost in some cases as these patients were either residing in far off areas or due to poor affordability of these patients. All patients with soft tissue and bony injuries were also followed in the plastic and orthopedic outpatient basis simultaneously. 30-days perioperative mortality was 5.48 %. The causes of mortality were disseminated intravascular coagulation (DIC) in 38.3 % (7), pulmonary embolism in 22.2 % (4) patients, cardiopulmonary arrest leading to myocardial infarction (MI) in 27.7 % (5) patients and 11.1 % (2) died because of renal failure.

## Discussion

Traumatic vascular injuries of the limbs remain a significant challenge especially in third world countries. In our retrospective analysis of the patients presenting with vascular trauma at a tertiary care centre, majority of the trauma is associated with blunt injuries mainly road traffic accidents followed penetrating injuries by firearms and bomb blasts. This study contrasts with the studies from west, as majority of the vascular injuries are caused by penetrating injuries due to civil violence [3] and decade long war situation in neighbouring country.

**Fig. 3:**
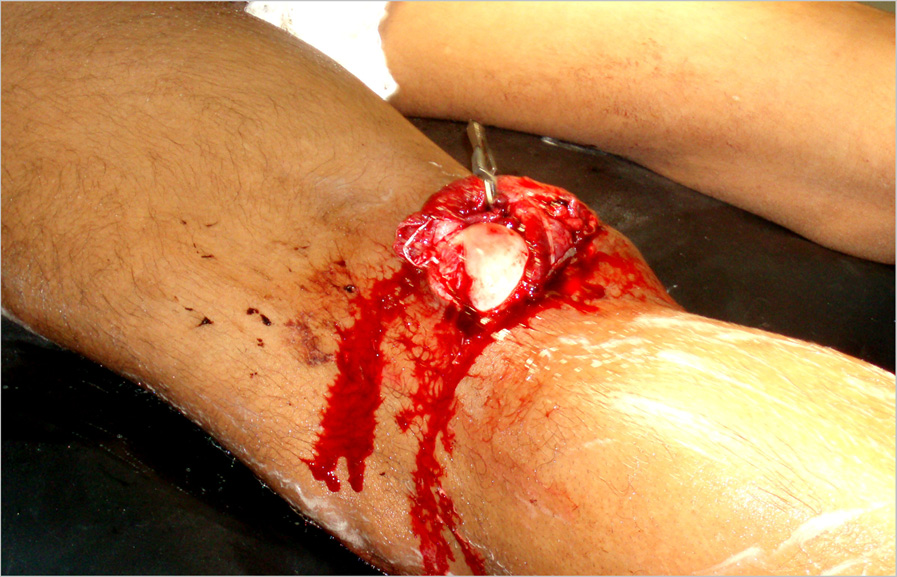
Left popliteal artery injury secondary to posteriorly dislocated distal femoral fracture in a victim of road traffic accident

The distribution of vascular trauma published by literature from European studies and trauma registries show a higher number of lower limb injuries as compared to upper limb [4] which is consistent with our study. The most common vascular injury in our series of patients is popliteal artery injury (Fig. 3). Injury of this vessel is regarded as the most challenging and threatening injury amongst all peripheral vascular injuries [5] and carries the greatest rate of limb loss [6]. Our centre receives patients from all parts of Pakistan and adjoining war afflicted areas such as Afghanistan. The scarcity of trauma surgeons’ especially vascular surgery services in remote areas and lack of effective ambulatory services in our country causes delay in the initial presentation of these patients. Time of presentation is a very essential prognostic marker of limb and overall survival [7]. Since the duration of presentation has a significant effect in delaying primary revascularization and limb loss [8], our study also shows that majority of the patients in whom the limb could not be salvaged presented after the crucial 8 hours of injury. Clinical examination was the initial approach among all the trauma patients and the use of contrast imaging modalities such as arteriography were reserved in cases with absent hard signs of vascular injury. Associated nerve and orthopaedic injuries contribute as added factor for morbidity and mortality in patients with vascular trauma as highlighted by McNamara et al [9] and by Desai P et al [10] too. In a study conducted by Topal AE showed that in patients presenting with trauma, 31.4 % associated bone injuries and 16.4 % had nerve injuries [11], as were found in our patients.

**Fig. 4:**
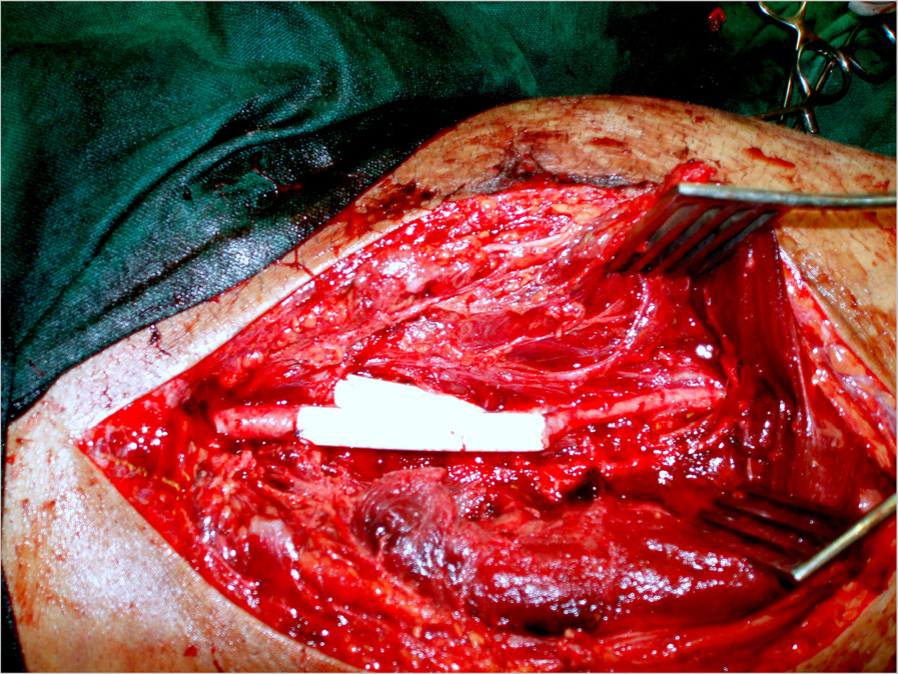
Arterial and venous repair was done in a patient with traumatic injuries involving femoral artery and vein

The choice of vascular repair depends upon the extent and severity of vascular trauma. Use of autogenous reverse vein graft as a conduit is the most common method of arterial repair in vascular trauma, which is supported from various studies in literature [12, 13]. In patients with associated venous injuries, venous repair was done for popliteal, femoral and subclavian veins [14] (Fig. 4), whereas rest of the veins were ligated. The delayed presentation resulting in long time interval between injury and reperfusion results in increase number of fasciotomies, as in our study 34.5 % had this accompanying procedure to relieve and prevent from the syndrome, which is observed by Menakuru SR et al. [15] too. Studies have shown that early closure of wound, within 72 hours of injury, results in lower complications and higher success rates, whereas vascular repairs that are exposed have a higher risk of wound desiccation, disruption of graft and secondary haemorrhage [16]. Soft tissue deficits were covered initially in patients who had extensive soft tissue injury. Besides surgery, use of endovascular techniques can be considered in carefully selected acute trauma patients [17]. This has a merit of providing access to remote areas, can be performed under local anaesthesia and reducing the risk of damage to surrounding structures. These options include balloon occlusion, percutaneous trans-catheter embolization and stent/stent graft placement [18].

Traumatic vascular injuries of the extremities are a major challenge especially in a third world country. Multiple factors involve in the prognosis of the affected limb. The most common factor involved is the delay in reducing ischemic reperfusion time. Other factor such as bone and nerve injuries and soft tissue deficits augments the chances of limb loss [19].

## Conclusion

With the rising trend of violence, terrorism and un-availability of facilities in developing countries, vascular injuries are becoming a major contributor of limb loss associated with increased morbidity and mortality. The most common factor is the delay in presentation to vascular services. Provision of proper training for dealing with trauma patients and evolving effective emergency ambulatory services are the key for reducing vascular injury related complications.
